# Hsc70 Focus Formation at the Periphery of HSV-1 Transcription Sites Requires ICP27

**DOI:** 10.1371/journal.pone.0001491

**Published:** 2008-01-30

**Authors:** Ling Li, Lisa A. Johnson, Jenny Q. Dai-Ju, Rozanne M. Sandri-Goldin

**Affiliations:** Department of Microbiology and Molecular Genetics, School of Medicine, University of California, Irvine, California, United States of America; University of Hong Kong, China

## Abstract

**Background:**

The cellular chaperone protein Hsc70, along with components of the 26S proteasome and ubiquitin-conjugated proteins have been shown to be sequestered in discrete foci in the nuclei of herpes simplex virus 1 (HSV-1) infected cells. We recently reported that cellular RNA polymerase II (RNAP II) undergoes proteasomal degradation during robust HSV-1 transcription, and that the immediate early protein ICP27 interacts with the C-terminal domain and is involved in the recruitment of RNAP II to viral transcription/replication compartments.

**Methodology/Principle Findings:**

Here we show that ICP27 also interacts with Hsc70, and is required for the formation of Hsc70 nuclear foci. During infection with ICP27 mutants that are unable to recruit RNAP II to viral replication sites, viral transcript levels were greatly reduced, viral replication compartments were poorly formed and Hsc70 focus formation was curtailed. Further, a dominant negative Hsc70 mutant that cannot hydrolyze ATP, interfered with RNAP II degradation during HSV-1 infection, and an increase in ubiquitinated forms of RNAP II was observed. There was also a decrease in virus yields, indicating that proteasomal degradation of stalled RNAP II complexes during robust HSV-1 transcription and replication benefits viral gene expression.

**Conclusions/Significance:**

We propose that one function of the Hsc70 nuclear foci may be to serve to facilitate the process of clearing stalled RNAP II complexes from viral genomes during times of highly active transcription.

## Introduction

The chaperone protein Hsc70 is part of a large group of proteins that assist in the folding and unfolding of proteins and in the assembly and disassembly of macromolecular structures [1]–[4]. In addition to protein folding, it has also been demonstrated that Hsc70 and an interacting co-chaperone U-box protein termed CHIP (C-terminus of Hsc70-interacting protein), target aberrant forms of CFTR (Cystic Fibrosis Transmembrane Conductance Regulator) protein for proteasomal degradation by promoting their ubiquitination [5]. Burch and Weller [6] reported that in herpes simplex virus 1 (HSV-1) infected cells, Hsc70 was sequestered in nuclear foci that also contained components of the 26S proteasome and ubiquitin-conjugated proteins. In subsequent studies, these authors also found that Hsp90 and Hsp40 were redistributed to these sites, which they termed VICE-domains, for virus-induced-chaperone-enriched [7]. The formation of these domains or foci at the periphery of viral replication compartments suggests that they may function to rid virus-infected nuclei of misfolded and ubiquitinated proteins via the proteasome, perhaps to prevent premature apoptosis, which would adversely affect viral replication.

We recently reported that during HSV-1 infection when viral transcription is robust, cellular RNA polymerase II (RNAP II), and specifically the serine-2 phosphorylated form of the C-terminal domain (CTD) found in elongating transcription complexes [8]–[10], undergoes ubiquitination and proteasomal degradation [11]. We speculated that this may occur because HSV-1 encodes transcripts from both genomic strands, and it encodes multiple sets of transcripts that initiate at the same promoter but have different polyadenylation sites or which initiate at different promoters but share a single polyadenylation site. At times of active transcription of all kinetic classes after DNA replication ensues, it is quite possible that elongating RNAP II complexes may collide if both strands are being transcribed at the same time, or may pile up on each other if 3′ processing lags behind elongation. It has been shown in yeast and mammalian cells that stalled RNAP II complexes become ubiquitinated and degraded by the proteasome to allow 3′ processing factors to access the RNA or to allow trailing transcription complexes to proceed [12]–[15]. We and others have shown that expression of the multifunctional regulatory protein ICP27 is essential for abundant transcription of HSV-1 early and late genes [16]–[23], and this is because ICP27 is required to recruit RNAP II to viral replication sites [11]. ICP27 interacts with RNAP II [24], and specifically with the CTD [11] and mutants of ICP27 that were not able to interact with RNAP II also were not able to recruit RNAP II to viral replication sites. In addition, viral replication compartment formation was greatly impaired, viral transcription was severely reduced, and proteasomal degradation of RNAP II was significantly diminished in infections with these ICP27 mutants [11].

Based upon the reported composition of the Hsc70 nuclear foci or VICE domains, we asked whether the formation of these discrete nuclear structures at the periphery of viral replication compartments was related to active viral transcription and RNAP II degradation. Here, we show ICP27 also interacts with Hsc70 and that Hsc70 nuclear focus formation required ICP27 expression. During infection with ICP27 mutants that cannot interact with RNAP II, viral transcription-replication compartments were poorly formed and Hsc70 focus formation was substantially delayed. Expression of a dominant negative Hsc70 mutant reduced RNAP II degradation, and this had adverse effects on viral replication. The results presented here lead us to postulate that Hsc70 foci serve an important role during viral infection, which includes, but is likely not limited to, aiding in the ubiquitination and proteasomal degradation of stalled RNAP II complexes on viral genomes.

## Results

### ICP27 Is Required for the Formation of Hsc70 Nuclear Foci

**Figure 1:**
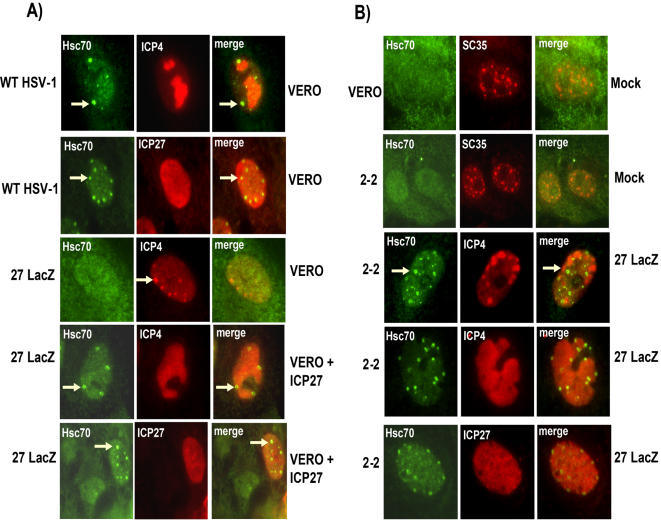
ICP27 Is Required for the Formation of Hsc70 Nuclear Foci. Vero cells or 2-2 cells were either mock-infected, infected with WT HSV-1 KOS or ICP27 null mutant 27-LacZ, or were first transfected with plasmid pSG130B/S, which expresses ICP27 [48], and were then infected 24 h later with 27-LacZ, as indicated. All infections were performed at a moi of 10. Cells were fixed at 6 h after infection and stained with anti-Hsc70, anti-SC35, anti-ICP4 and anti-ICP27 antibodies as indicated. Antibody to the nuclear splicing SR protein SC35 was used in the mock-infected sample to define the nucleus. Arrows mark sites of Hsc70 nuclear foci (green) or ICP4-containing pre-replication sites (red). In this figure, and in all subsequent immunofluorescence images, representative cells are shown at higher magnification to better illustrate the pattern of protein localization and to observe intracellular structures. In all cases, cells shown in the figures represent the pattern observed in greater than 75% of the cells visualized in at least 10 fields.

To determine if ICP27 played any role in the formation of Hsc70 nuclear foci, as has been shown for the HSV-1 immediate early protein ICP0 [6], we analyzed the distribution of Hsc70 in wild type HSV-1-infected cells compared to cells infected with 27-LacZ, an ICP27 null mutant virus. In wild type HSV-1 infected cells at 6 h after infection, staining of Hsc70 was seen to concentrate in distinct nuclear foci, which occurred at the periphery of viral transcription-replication compartments. These compartments were marked by staining with antibody to the HSV-1 transcriptional activator ICP4 (Figure 1). In contrast, there was a diffuse nuclear and cytoplasmic distribution of Hsc70 staining in 27-LacZ-infected cells, which was similar to what was seen in mock-infected cells (Figure 1). Further, viral transcription-replication compartments were poorly formed. However, 27-Lac-Z infection of the complementing cell line, 2-2, which expresses ICP27 in *trans* upon infection [25], resulted in the formation of Hsc70 nuclear foci. Similarly, in Vero cells that were transfected with a plasmid expressing ICP27, and which were then infected with 27-LacZ, the formation of Hsc70 nuclear foci was seen at the periphery of viral transcription-replication compartments (Figure 1). Further, Hsc70 focus formation was seen in a cell expressing ICP27, but not in the cell next to it, which did not express ICP27 (Figure 1, bottom left panels). It should also be noted that ICP27 expression was seen to be at similar levels in the 27-LacZ-infected 2-2 cells and in the transfected Vero cells compared to WT infection (Figure 1). We conclude that ICP27 expression is necessary for the formation of Hsc70 nuclear foci during HSV-1 infection.

### Both the N- and C-termini of ICP27 Are Required to Interact with Hsc70 In Vitro and In Vivo

**Figure 2:**
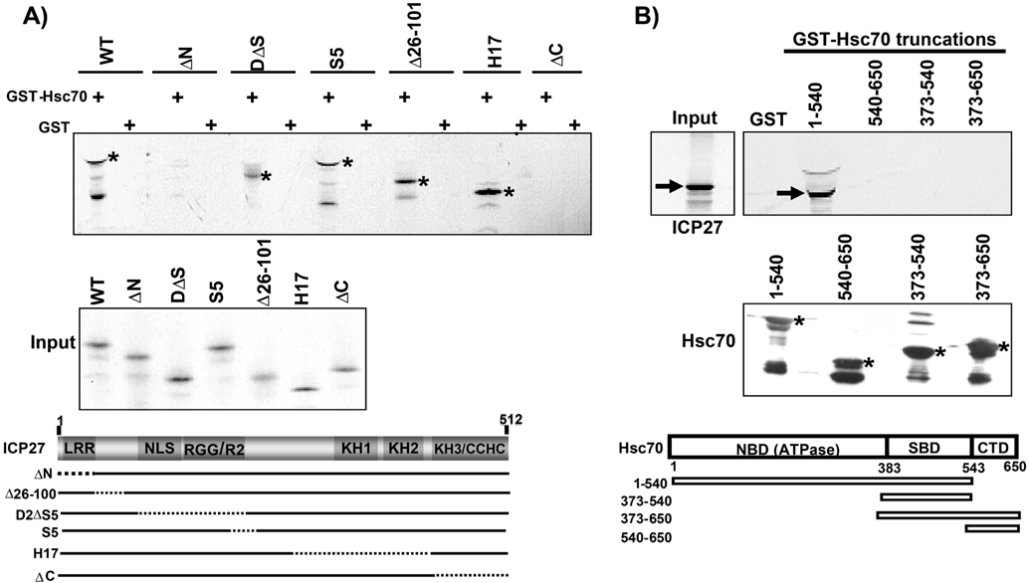
ICP27 Associates with Hsc70 *in vitro.* A) GST-binding assays were performed with GST-Hsc70 (1-540) and in vitro translated WT ICP27 and mutants ΔN, D2ΔS5, S5, Δ26-100, H17 and ΔC. Asterisks mark the position of the ICP27 proteins that were seen to bind. Input ^35^S-labeled proteins are shown in the middle panel. In the bottom panel, a schematic diagram of the 512 amino acid ICP27 coding region, including the leucine-rich amino terminus (LRR), nuclear localization signal (NLS), RGG box RNA binding motif (RGG), arginine-rich region (R2), three predicted KH domains and a zinc-finger-like motif (CCHC) in the C-terminus. The positions of the deletion mutations (dotted lines) are shown below. B) GST binding assays were performed with ^35^S-labeled, in vitro-translated WT ICP27 and GST-tagged Hsc70 truncated proteins. The arrow shows the position of ICP27. Input GST-Hsc70 proteins are shown in the middle panel. A schematic of the Hsc70 coding region is shown illustrating the nucleotide binding domain (NBD-ATPase), the substrate binding domain (SBD) and the C-terminal domain (CTD).

ICP27 is a highly interactive protein that has been shown to interact with itself to form multimers [26] and with a number of cellular proteins, including RNAP II [11], [24], splicing factors [27], [28], and mRNA export factors Aly/REF and TAP/NXF1 [29]–[31]. To determine if ICP27 interacts with Hsc70, in vitro binding assays were performed using bacterially-expressed GST-Hsc70 protein encoding amino acids 1 to 540. This includes the Nuclear Binding Domain (NBD- ATPase) and the Substrate Binding Domain (SBD) but not the C-terminal Domain (CTD). In vitro translated, ^35^S-methionine-labeled wild type and mutant versions of ICP27 were used in these binding assays (Figure 2). WT ICP27 and deletion mutants DΔS5, S5, Δ26-101 and H17 were seen to bind to GST-Hsc70; however, ΔN, in which amino acids 3 to 28 are deleted, and ΔC, in which residues 450 to 512 are deleted, failed to bind to Hsc70 (Figure 2A). These results indicate ICP27 binds Hsc70 in vitro and that both the N- and C-termini of ICP27 must be intact for interaction. A similar result was found for the interaction of ICP27 with RNAP II CTD and with TAP/NXF1 [11], [30], that is, both the N- and C-termini were required for interaction. In assays with Hsc70 truncation mutants, only the 1-540 species of Hsc70 bound to ICP27 (Figure 2B), indicating that the ATPase domain and the substrate binding domain of Hsc70 are required for interaction with ICP27 but not the CTD.

**Figure 3:**
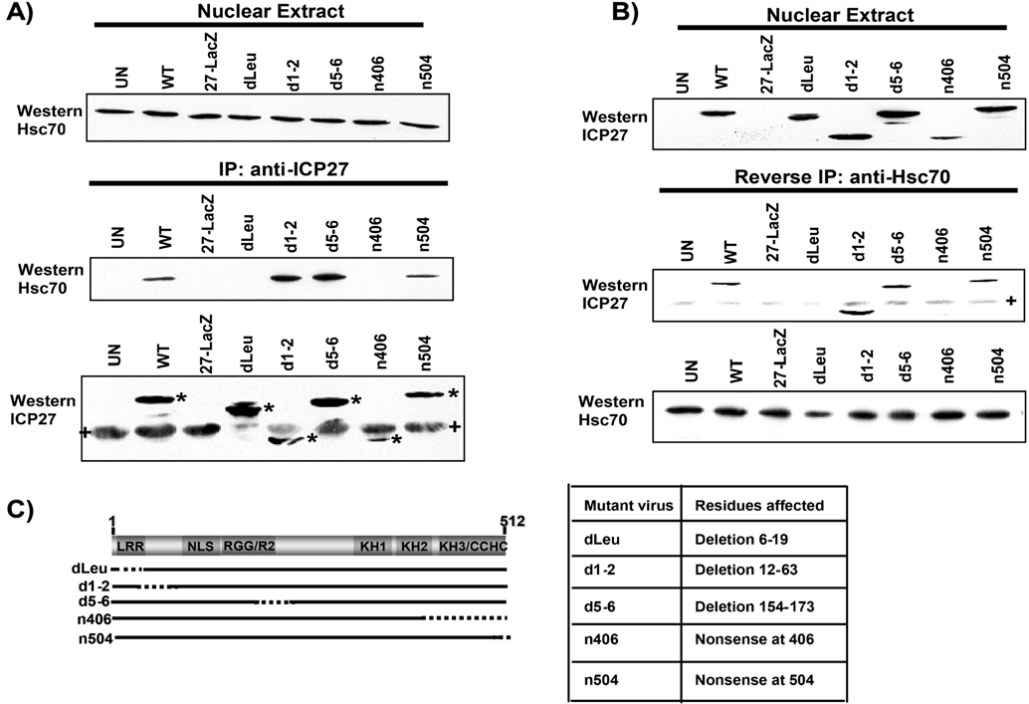
The N- and C-termini of ICP27 Are Required for Interaction with Hsc70 during Infection. A) HeLa cells were either mock-infected or infected with WT HSV-1 or ICP27 mutants as indicated. At 6 h after infection, nuclear extracts were prepared. Expression of endogenous Hsc70 in mock-infected cells and HSV-1 WT- and mutant-infected cells is shown by Western blot analysis of samples from nuclear extracts in the top panel. Immunoprecipitations with anti-ICP27 antibody followed by Western blot analysis with anti-Hsc70 antibody are shown in the middle panel. The bottom panel shows a Western blot of anti-ICP27 immunoprecipitated samples probed with anti-ICP27 antibody to show ICP27 expression. Asterisks (*) mark WT and mutant ICP27 protein bands. The + signs mark heavy chain IgG from the immunoprecipitations. B) Reverse immunoprecipitations were performed on samples from the nuclear extracts described above. The top panel shows the expression of WT and mutant ICP27 in the nuclear extracts. Immunoprecipitation of each sample was performed with anti-Hsc70 antibody and Western blot analysis was performed by probing with anti-ICP27 antibody, as shown in the middle panel. The + sign marks heavy chain IgG from the immunoprecipitation. The bottom panel shows a Western blot of anti-Hsc70 immunoprecipitated samples probed with anti-Hsc70 antibody. C) A schematic diagram of the ICP27 coding region is shown in the left panel with the positions of the deletions illustrated by dotted lines. The table in the right panel describes the residues deleted or mutated in each mutant virus.

To confirm the interaction of ICP27 with Hsc70 in virus-infected cells, co-immunoprecipitation experiments were performed. Cells were infected with WT HSV-1 strain KOS or with ICP27 mutant viruses as indicated (Figure 3) and at 6 hours after infection, cell lysates were immunoprecipitated with anti-ICP27 monoclonal antibody. Western blot analysis was performed with anti-Hsc70 antibody. Hsc70 was co-immunoprecipitated with ICP27 in WT HSV-1-infected cells and in cells infected with mutants d1-2, d5-6 and n504 (Figure 3A). Hsc70 was not detected in immunoprecipitated samples from cells infected with the null mutant 27-LacZ, the N-terminal mutant dLeu, nor the C-terminal mutant n406. Similarly, in the reverse immunoprecipitation experiment performed with anti-Hsc70 antibody, ICP27 was not detected in Hsc70 immunoprecipitated samples from 27-LacZ-, dLeu- and n406-infected cells (Figure 3B). We conclude that ICP27 interacts with Hsc70 in virus-infected cells and N-terminal residues 6-19 and C-terminal residues 406 to 504 must be intact for this interaction to occur.

### Hsc70 Nuclear Sequestration Is Delayed in Infections with N- and C-terminal ICP27 Mutants

**Figure 4:**
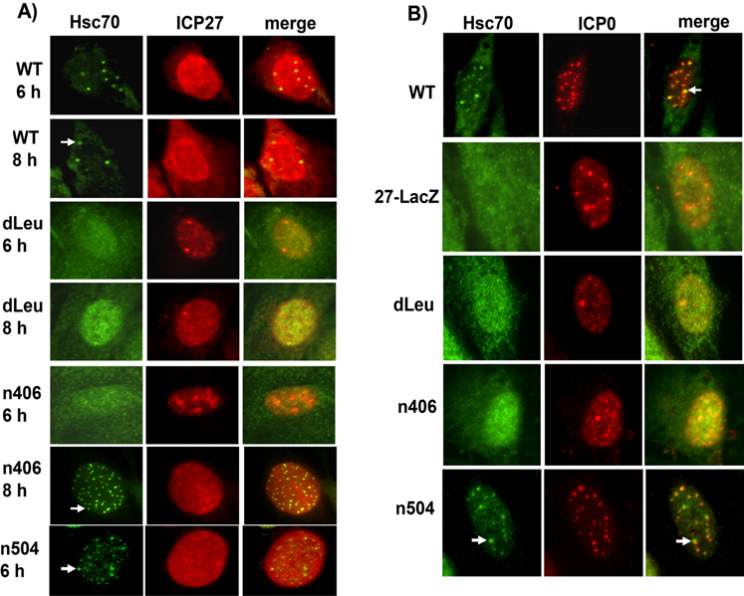
Hsc70 Nuclear Focus Formation Is Delayed in Infections with ICP27 Mutants. A) Vero cells were infected with WT HSV-1 or mutants dLeu, n406 or n504. At the times indicated, cells were fixed and stained with anti-Hsc70 and anti-ICP27 antibodies as indicated. Arrows mark Hsc70 nuclear foci. B) Vero cells were infected with WT HSV-1, 27-LacZ, dLeu, n406 or n504 for 6 h, at which time cells were fixed and stained with anti-Hsc70 and anti-ICP0 antibodies. The arrow marks an Hsc70 focus adjacent to an ICP0 speckle.

To determine if Hsc70 was relocalized to nuclear foci during infection with ICP27 mutants that do not interact with Hsc70, immunofluorescent staining was performed. In cells infected with WT HSV-1, Hsc70 focus formation was clearly seen by 6 h after infection, a time when ICP27 is still strongly nuclear, but it has begun to shuttle to the cytoplasm in its role as a viral RNA export factor (Figure 4A). The shuttling of ICP27 can be seen more clearly at 8 h after infection (Figure 4A). In contrast, in dLeu-infected cells, Hsc70 foci were not observed even by 8 h after infection; while in n406-infected cells, small Hsc70 foci were seen but not until 8 h after infection, when larger foci are apparent in WT-infected cells (Figure 4A). Because viral replication is both curtailed and delayed in n406 infected cells, the smaller foci seen at 8 h in n406 infections, probably reflect the timing of the beginning of DNA replication. Further, both dLeu and n406 are confined to the nucleus because these ICP27 mutants do not interact with TAP/NXF, which is required for ICP27 to shuttle to the cytoplasm [29]. Interestingly, mutant n504, which also does not interact with TAP/NXF1 was confined to the nucleus at 6 h after infection, however, Hsc70 foci were seen to form. This mutant does interact with Hsc70.

Burch and Weller [6] reported that the HSV-1 immediate early protein, ICP0, which encodes an E3-ubiquitin ligase in its RING domain [32], was necessary to establish Hsc70 foci at early times during infection and was sufficient to redistribute chaperone molecules in transfected cells. Immunofluorescent staining of ICP0 and Hsc70 in WT and ICP27 mutant infected cells showed that ICP0 was similarly expressed in a nuclear punctate staining pattern at 6 h after infection; however, Hsc70 foci were only observed in WT HSV-1-infected cells (Figure 4B). These results corroborate the results shown in Figure 1 indicating that ICP27 is also required for the nuclear sequestration of Hsc70 in HSV-1-infected cells.

### Hsc70 Focus Formation Correlates with HSV-1 Transcription-Replication Compartment Formation

**Figure 5:**
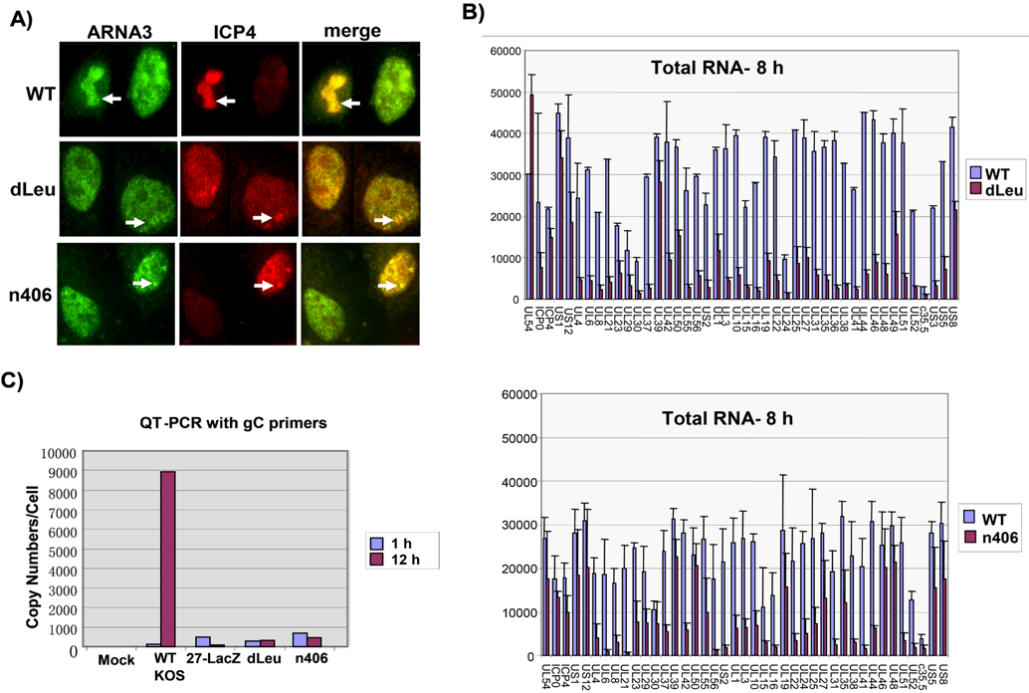
ICP27 N- and C-Terminal Mutants Show Poor Replication Compartment Formation and Reduced Transcription and DNA Replication. A) Vero cells were infected with WT HSV-1, dLeu or n406 for 8 h, at which time cells were fixed and stained with anti-RNAP II antibody ARNA3, which recognizes all forms of RNAP II, and anti-ICP4 antibody. The arrows point to a fully formed replication compartment in WT-infected cells and to small pre-replication sites in dLeu and n406-infected cells. B) HeLa cells were infected with WT HSV-1, dLeu or n406 as indicated. At 8 h after infection total RNA was extracted and microarray analysis was performed as described previously against an array of HSV-1 transcript-specific probes [35]. Hybridizations were performed in triplicate and the experiments were performed twice. Error bars represent the standard deviations. C) Vero cells were mock-infected or were infected with WT HSV-1, 27-LacZ, dLeu or n406 at a moi of 10. At 1 h and 12 h after infection, DNA was purified from cell lysates by phenol-chloroform extraction. Quantitative real-time PCR was performed with a probe for the glycoprotein C (gC) gene to determine viral DNA copy number.

Viral transcription and replication is greatly reduced during infection when ICP27 is not expressed, as in 27-LacZ infection, and in infections with ICP27 mutants that cannot interact with RNAP II to recruit it to viral replication sites [11], [18], [23], [33], [34]. Mutants dLeu and n406 do not interact with RNAP II [11]. As a result, RNAP II was not recruited to sites of viral replication, marked by staining for ICP4, and replication compartments were poorly formed (Figure 5A, compare WT to dLeu and n406). The inefficient recruitment of RNAP II to viral replication sites is the likely cause of the low levels of early and late viral transcripts produced during infection with these mutants compared to WT, as shown by microarray analysis (Figure 5B) using an array of HSV-1 DNA probes that span the genome, as described previously [35]. The low levels of viral transcripts in turn results in very low levels of DNA replication for these mutants as shown by quantitative PCR with a probe for the viral glycoprotein C (gC) gene, which resides in the unique long region of the viral genome. While viral DNA copies per cell were amplified about 9000 fold in WT HSV-1 infected cells by 12 h after infection, little increase in DNA copy number was seen in mutant infected cells (Figure 5C).

**Figure 6:**
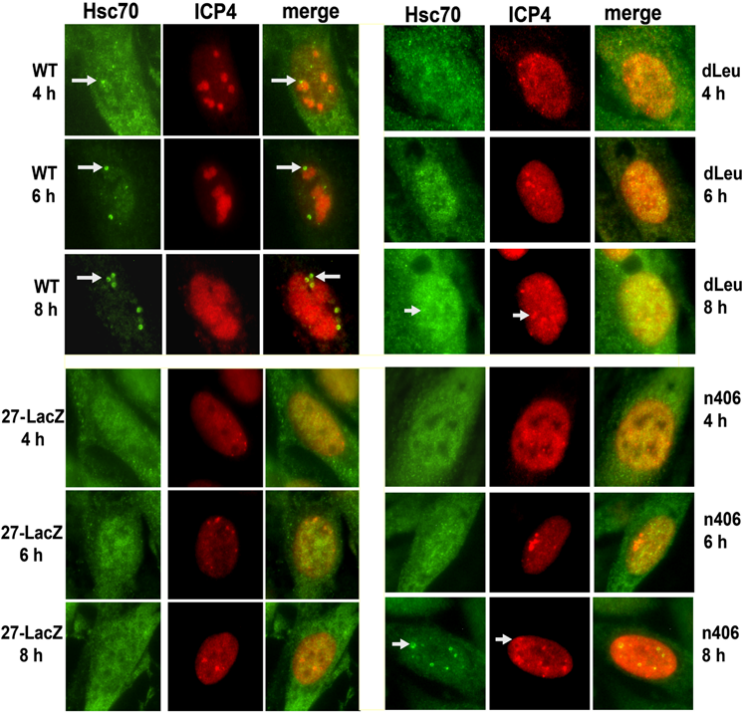
Hsc70 Focus Formation Correlates with HSV-1 Replication Compartment Formation. Vero cells were infected with WT HSV-1, 27-LacZ, dLeu or n406. At the times indicated, cells were fixed and stained with anti-Hsc70 and anti-ICP4 antibodies. Arrows point to Hsc70 foci (green) or pre-replication sites (red).

Next, we analyzed Hsc70 nuclear focus formation and viral replication compartment formation in ICP27 mutant virus-infected cells compared to WT at different times after infection. While Hsc70 foci were seen to form adjacent to pre-replication compartment structures by 4 h after infection in WT-infected cells (Figure 6), there was little evidence of Hsc70 foci even by 8 h in 27-Lac Z and dLeu-infected cells, and viral replication compartment formation was also greatly compromised. Small foci of Hsc70 were seen in n406-infected cells but not until 8 h after infection, when ICP4 containing pre-replication sites could be seen (Figure 6). Thus, it appears that Hsc70 nuclear sequestration and focus formation correlates with the formation of HSV-1 replication compartments and Hsc70 nuclear foci are most apparent with fully formed viral replication compartments.

### Hsc70 Foci Arise Concomitantly with the Loss of Serine-2 phosphorylated RNA Polymerase II

A unique feature of HSV-1 transcription is that there is decrease in the serine-2 phosphorylated form of the RNAP II CTD, which is found in RNAP II elongating complexes [11], [36], [37]. This is accompanied by an overall decrease in RNAP II protein levels at later times during infection when viral transcription is very robust. This decrease results from proteasomal degradation of RNAP II because it can be prevented by adding the proteasome inhibitors MG132 or lactacystin [11]. During infections with ICP27 mutants that fail to interact with RNAP II, viral transcription is greatly reduced and delayed and so is the degradation of RNAP II [11]. Thus, we concluded previously that phospho-serine-2 RNAP II degradation most likely occurred during highly active viral transcription and that it resulted from elongating complexes stalling in high traffic areas of the genome [11]. Proteasomal degradation would clear the stalled complexes. Therefore, we next sought to determine if Hsc70 focus formation was tied to efficient recruitment of RNAP II to viral replication sites, resulting in robust transcription, which could then lead to RNAP II degradation. Hsc70 nuclear structures have been reported to contain components of the proteasomal machinery [6], and therefore the localization of these foci at the periphery of viral transcription-replication sites may serve an important function in facilitating ubiquitination and proteasomal degradation of stalled RNAP II complexes.

**Figure 7:**
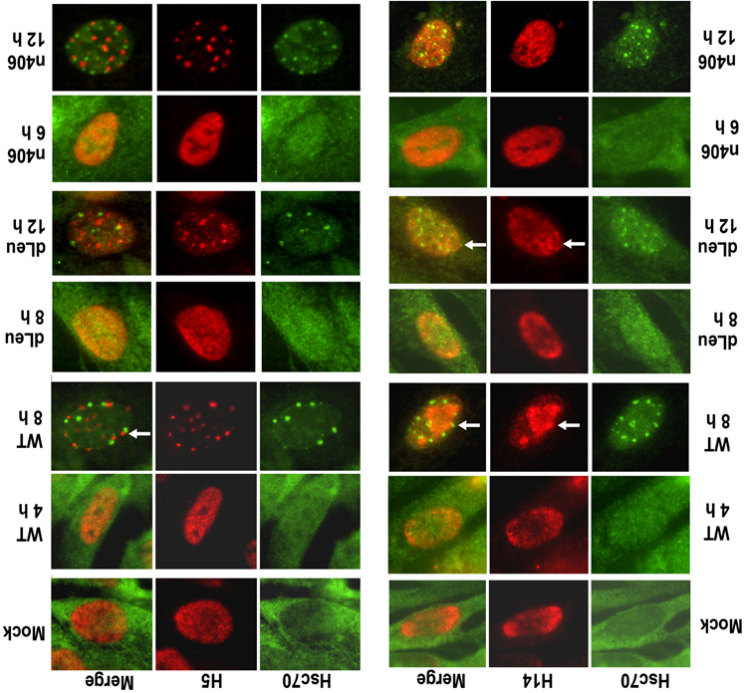
Hsc70 Nuclear Sequestration Correlates with the Loss of Phospho-serine 2 Form of RNAP II CTD in Fully Formed Replication Compartments. Vero cells were mock-infected or infected with WT HSV-1, dLeu or n406 for the times indicated. In the left hand panels, cells were stained with anti-RNAP II antibody, H14, which recognizes phospho-serine 5 RNAP II CTD, and anti-Hsc70 antibody. Arrows show a replication compartment with an Hsc70 focus site at the periphery in the 8 h WT-infected cell, and a pre-replication site (red) next to a small Hsc70 focus site (merge) in the 12 h dLeu-infected cell. In the right panels, cells were stained with anti-Hsc70 antibody and anti-RNAP II antibody H5, which recognizes phospho-serine-2 RNAP II CTD. This antibody also cross-reacts with a phospho-epitope in splicing SR proteins [11], [40]. The arrow points to an Hsc70 focus site (green) adjacent to a splicing speckle (red).

To monitor relocalization of RNAP II to viral replication compartments, staining was performed with monoclonal antibody H14, which recognizes the serine-5 phosphorylated form of RNAP II CTD found in the initiation complex [38], [39]. Serine-5 phosphorylated RNAP II was recruited to structures resembling viral replication compartments by 8 h after infection in WT HSV-1-infected cells, and Hsc70 foci formed at the periphery of these structures (Figure 7, left panels). To show that these structures were indeed replication compartments, H14 stained cells were also stained with antibody to ICP4 (Figure S1). It is clear that serine-5 RNAP II stained by H14 antibody colocalized with ICP4-marked replication compartments. In contrast, in dLeu and n406 infections, H14 staining was similar to mock infected cells at 8 h for dLeu and 6 h for n406-infected cells (Figure 7, left panels). It was not until later times with these mutants that small Hsc70 foci were seen to form along with small compartments that stained with H14, and which presumably represent viral pre-replication sites (Figure 7, left panels and Figure S1).

Degradation of the serine-2 phospho-form of RNAP II CTD was assessed by staining with monoclonal antibody H5 (Figure 7, right panels), which recognizes the serine-2 phosphorylated CTD [38], [39]. We showed previously that along with proteasomal degradation of RNAP II during HSV-1 infection, there is a loss of H5 staining and the subset of H5 stained structures that remain have a speckled appearance [11], because this antibody cross-reacts with a phospho-epitope of SR protein splicing factors [40]. Fraser and Rice [36] reported a similar finding in HSV-1 infected cells. The cross-reactivity of H5 with SR proteins occurs when levels of serine-2 phosphorylated RNAP II CTD are low and SR proteins are far more abundant [40]. Thus, RNAP II degradation can be monitored by the change in the staining pattern seen with H5 antibody [11], [36], [37]. In mock infected cells, H5 staining is diffuse throughout the nucleus, and this is also seen at 4 h in WT HSV-1 infected cells (Figure 7, right panels). However, by 8 h, H5 staining was greatly reduced and the staining was now confined to speckled structures (Figure 7 and Figure S1). That these speckled structures were splicing factors was confirmed by staining with a monoclonal antibody to SR protein SC35 (Figure S2). Further, Hsc70 focus formation correlated with the appearance of H5 speckles, and the Hsc70 foci were adjacent to the speckles (Figure 7, right panels, and Figure S2, lower panel).

In dLeu and n406 infections, the appearance of Hsc70 nuclear foci, and H5 speckled staining was greatly delayed and was not apparent until 12 h after infection (Figure 7, right panels, and Figure S2, middle panels). This is in accord with the reduced and delayed viral transcription pattern seen in infections with these mutants (Figure 5), and a resultant delay in RNAP II degradation [11]. Therefore, the appearance of Hsc70 nuclear foci at the periphery of viral transcription/replication sites may occur as a result of the ubiquitination of RNAP II on stalled elongating complexes in high traffic areas of an actively replicating viral genome.

### Preventing RNAP II Degradation Also Prevents Hsc70 Focus Formation

**Figure 8:**
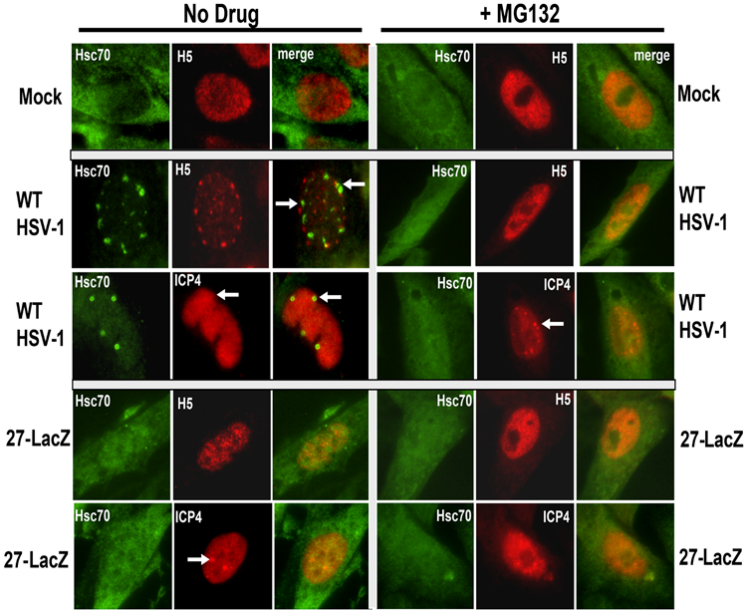
Preventing Phospho-serine 2 RNAP II Degradation Also Prevents Hsc70 Focus Formation. Vero cells that were mock-infected or were infected with WT HSV-1 or 27-LacZ were untreated (left panels) or were treated with 50 µM MG132 added 1 h after infection (right panels). Cells were fixed at 8 h after infection and stained with anti-Hsc70 antibody, antibody H5 and anti-ICP4 antibody as indicated. Arrows mark Hsc70 foci (green) or replication or pre-replication sites (red).

The degradation of RNAP II during HSV-1 infection can be prevented by addition of the proteasomal inhibitor MG132 [11]. We asked what effect MG132 would have on Hsc70 focus formation. There was no change in the diffuse nuclear and cytoplasmic staining of Hsc70 in mock and 27-LacZ-infected cells with or without MG132 (Figure 8). In contrast, Hsc70 nuclear focus formation was curtailed in WT HSV-1-infected cells in the presence of MG132, as was the appearance of H5 speckled structures (Figure 8). Therefore, preventing the proteasomal degradation of RNAP II during HSV-1 infection also precludes the formation of Hsc70 nuclear foci.

### An Hsc70 Dominant Negative Mutant Can Prevent RNAP II Degradation and Focus Formation

A loss of function mutant of Hsc70, which has a methionine substituted for lysine 71 in the ATPase domain, has been shown to be unable to hydrolyze ATP, which is essential for Hsc70 function [41]. Mutant Hsc70 K71M behaves in a dominant negative fashion when expressed in cultured cells, in that it interferes with endogenous Hsc70 function [1], [41], [42]. To determine first if Hsc70 K71M was capable of interacting with ICP27, cells were transfected with GFP-tagged Hsc70 K71M and with GFP-tagged Hsc70 as a control. Cells were infected with WT HSV-1 and co-immunoprecipitations were performed on cell lysates. In samples that were immunoprecipitated with antibody against ICP27, GFP-Hsc70 was co-immunoprecipitated, as shown by probing a Western blot with antibody to GFP (Figure S3, middle panel). GFP-Hsc70 K71M was not found to be precipitated with ICP27. In re-probing the same blot with antibody against Hsc70, it was seen that endogenous Hsc70 was also present in the immunoprecipitated ICP27 complexes in control transfections with GFP and in transfections with GFP-Hsc70 (Figure S3, right panel). However, endogenous Hsc70 was not seen in samples from cells expressing GFP-Hsc70 K71M, the dominant negative mutant. This indicates that not only does Hsc70 K71M fail to interact with ICP27, but also that it appears to interfere with the interaction of endogenous Hsc70 and ICP27.

**Figure 9:**
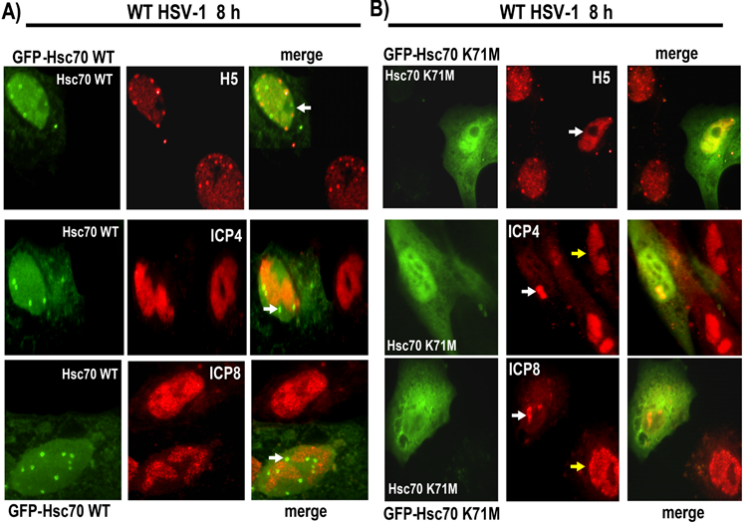
An Hsc70 Dominant Negative Mutant Can Prevent RNAP II Phospho-serine 2 Degradation and Hsc70 Focus Formation. A) Vero cells were transfected with pGFP-Hsc70 and were subsequently infected with WT HSV-1 for 8 h. Cells were stained with H5, anti-ICP4 or anti-ICP8 antibodies. GFP fluorescence was viewed directly. Arrows point to Hsc70 foci. B) Vero cells were transfected with the dominant negative mutant GFP-Hsc70K71M and then infected with WT HSV-1 for 8 h. Cells were stained with H5, anti-ICP4 or anti-ICP8 antibodies. Arrows point to GFP Hsc70K71M expressing cells showing diffuse nuclear staining with H5, and to pre-replication sites in cells stained for ICP4 and ICP8. The yellow arrows in the ICP4 and ICP8 stained panels point to a fully formed replication compartment.

To determine if Hsc70 K71M would interfere with Hsc70 focus formation during HSV-1 infection, cells transfected with GFP-Hsc70 K71M or GFP-Hsc70 were subsequently infected with WT HSV-1 for 8 h. Hsc70 localization was monitored by immunofluorescent microscopy. In cells transfected with GFP-Hsc70, nuclear focus formation was seen, along with speckled staining for H5, and replication compartment formation marked by staining for ICP4 and ICP8, a viral DNA replication protein (Figure 9A). In contrast, in cells expressing GFP-Hsc70 K71M, there was a diffuse nuclear and cytoplasmic distribution with no focus formation. Even more interesting was the staining of H5 in the cell expressing GFP-Hsc70 K71M, which remained diffuse in the nucleus rather than localizing to speckles as in the infected cells not expressing GFP-Hsc70 K71M (Figure 9B). This suggests that degradation of RNAP II was not occurring in the presence of the dominant negative mutant. Further, viral replication compartment formation was also hampered by the dominant negative Hsc70 mutant, as seen in the cells stained with ICP4 and ICP8 antibodies, and which also expressed GFP-Hsc70 K71M (Figure 9B).

**Figure 10:**
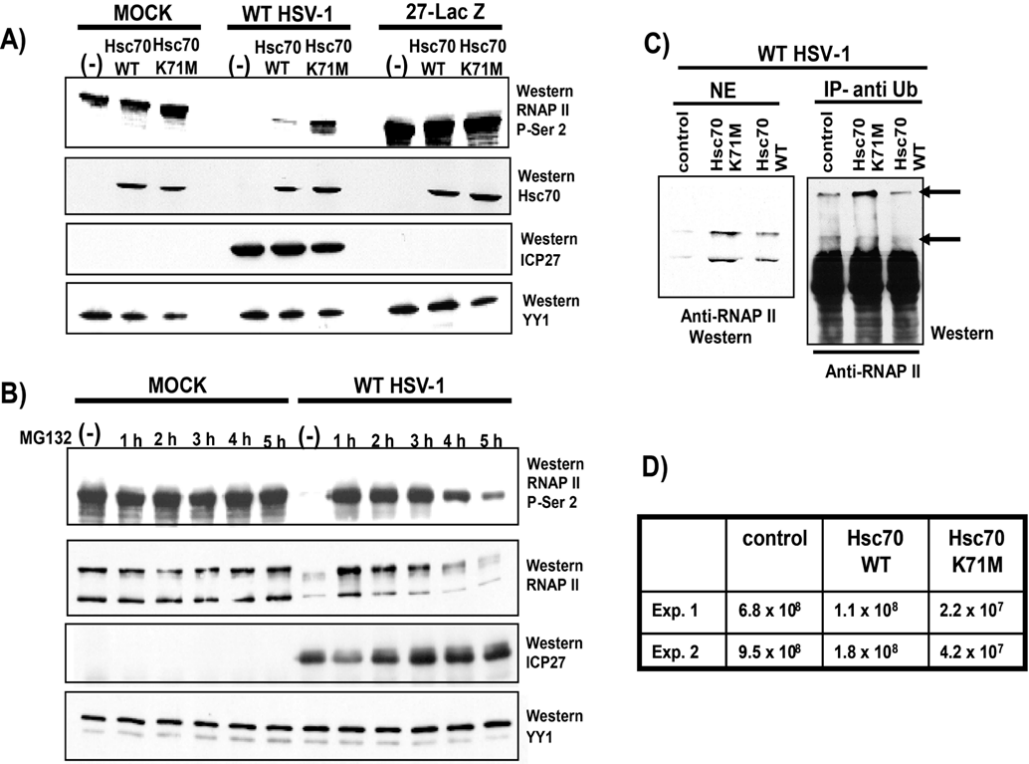
Dominant Negative Mutant Hsc70 K71M Prevents Proteasomal Degradation of Phospho-Serine 2 RNAP II CTD. A) RSF cells were transfected with vector alone (-), Myc-tagged Hsc70 or Myc-tagged Hsc70K71M and 24 h later were either mock-infected or were infected with WT HSV-1 or 27-LacZ. Nuclear extracts were prepared 8 h after infection. Western blots were probed with antibody H5 to detect the phospho-serine 2 form of RNAP II; anti-myc antibody to detect myc-tagged Hsc70; anti-ICP27 antibody to monitor ICP27 expression, and anti-YY1 antibody as a loading control. YY1 is a cellular transcription factor. B) HeLa cells were either mock-infected or infected with WT HSV-1. MG132 (50 µM) was added at the times indicated and nuclear extracts were prepared at 8 after infection. Western blots were probed with H5 antibody to detect RNAP II phospho-serine 2; ARNA3 antibody, which recognizes all forms of RNAP II, both phosphorylated and unphosphorylated; anti-ICP27 antibody, and anti-YY1 antibody. C) RSF cells were transfected with empty vector alone (control), myc-tagged Hsc70 or myc-tagged Hsc70K71M as indicated, and were infected 24 h later with WT HSV-1. Nuclear extracts were prepared 8 h after infection, and samples were fractionated directly by SDS-PAGE for Western blot analysis (left panel, NE) or were immunoprecipitated with anti-ubiquitin antibody (right panel, IP- anti-Ub). Immunoprecipitated complexes were fractionated by SDS-PAGE and the Western blot was probed with anti-RNAP II antibody ARNA3. Arrows point to slower migrating ubiquitinated forms of RNAP II. D) RSF cells were transfected with empty vector alone (control), myc-tagged Hsc70 or myc-tagged Hsc70K71M and were infected 24 h later with WT HSV-1. Cells were harvested 24 h after infection and viral titers were determined by plaque assays.

To further explore the apparent inhibition of RNAP II degradation by the Hsc70 dominant negative mutant, Western blot analysis was performed on nuclear extracts from cells that were transfected with GFP or GFP-Hsc70 as controls, or with GFP-Hsc70 K71M. Cells were subsequently mock-infected or were infected with WT HSV-1 or 27-LacZ. Blots were probed with antibody H5 to detect the serine-2 form of RNAP II CTD. Note that in Western blots, cross-reactivity of H5 with SR proteins does not prevent detection of serine-2 RNAP II CTD because its apparent molecular weight is greater than 180 kDa, whereas, the cross-reacting SR proteins are around 35 kDa. Levels of serine-2 phosphorylated RNAP II CTD were similar in all samples from mock infected cells and in all samples from cells infected with 27-LacZ (Figure 10A). This is in accord with our previous finding that there is no discernible loss of the serine-2 form in mock infected cells, and that degradation of RNAP II occurs to a lesser extent and at much later times in 27-LacZ infected cells, which are severely defective in viral transcription [11], [43]. On the other hand, there was a marked reduction in the serine-2 form of RNAP II in WT HSV-1 infected cells, such that it was not detectable in cells transfected with GFP alone and was barely detectable in cells transfected with GFP-Hsc70, indicating that proteasomal degradation had occurred. The dominant negative Hsc70 K71M interfered with RNAP II degradation, because levels of the serine-2 phospho-form that were detected were significantly higher compared with the controls (Figure 10A). The rescue of RNAP II from degradation by Hsc70 K71M was on par with the addition of MG132 (Figure 10B). When MG132 was added to WT HSV-1 infected cells at different times after infection, there was inhibition of phosphoserine-2 RNAP II and total RNAP II degradation, with the greatest effects seen with the early addition of the drug before robust viral transcription and replication (Figure 10B).

We previously reported that RNAP II undergoes ubiquitination during HSV-1 infection, and that higher levels of ubiquitinated species were seen in the presence of MG132, because degradation by the proteasome was precluded [11]. To determine if the dominant negative Hsc70 mutant could similarly trigger an increase in ubiquitinated RNAP II, immunoprecipitation with anti-ubiquitin antibody was performed on nuclear extracts from HSV-1 infected cells that had been transfected with GFP or GFP-Hsc70 as controls or GFP-Hsc70 K71M. Western blots were then probed with an antibody that recognizes all forms of RNAP II, both phosphorylated and unphosphorylated. Higher migrating bands were detected and the intensity of these bands was greater in the sample from cells expressing the dominant negative Hsc70 mutant (Figure 10C). In addition, it can be seen from the nuclear extract samples that total RNAP II levels were reduced in the control GFP and GFP-Hsc70 samples, compared to the GFP-Hsc70 K71M sample (Figure 10C, left panels). These results indicate that the dominant negative Hsc70 mutant interferes with the degradation of ubiquitinated RNAP II. We showed previously [11] that prevention of RNAP II degradation by MG132 or lactacystin results in a reduction in viral yields. We also saw a decrease in virus yield in cells transfected with the Hsc70 K71M mutant (Figure 10D). Although yields were reduced only about 20 fold, this may be an underestimate of the effect because only a subset of the infected cells was successfully transfected with the dominant negative mutant plasmid. Thus, we conclude that clearing of stalled RNAP II complexes during robust transcription and DNA replication is beneficial for HSV-1 infection, and preventing proteasomal degradation is detrimental.

## Discussion

It has been reported that RNAP II elongating complexes that stall on DNA templates due to DNA damage or to a build up of elongating complexes in highly transcribed genes are subject to ubiquitination and proteasomal degradation [13], [15], [44], [45]. We have reported that during HSV-1 infection, RNAP II becomes ubiquitinated and undergoes proteasomal degradation that can be prevented by the addition of MG132 and lactacystin [11]. The phospho-serine 2 form of the CTD, found in the elongating complex, is most severely affected. Further, prevention of RNAP II degradation by proteasome inhibitors resulted in decreased levels of viral late proteins and reduced viral yields [11]. The degradation of RNAP II occurred earlier in infection and was more profound in wild type HSV-1 infected cells compared to infections with viral mutants that either did not express ICP27 or in which the mutant ICP27 was unable to interact with and recruit RNAP II to viral replication sites [11]. These mutants accumulate low levels of viral transcripts and have greatly reduced DNA replication ([11] and Figure 5). These results led us to postulate that when HSV-1 genomes are undergoing robust transcription and DNA replication, elongating RNAP II complexes may collide, especially in regions of the genome where transcripts are being transcribed on both strands; in regions where several transcripts share the same polyadenylation sites, and in regions where the 3′ end of a gene is juxtaposed or even overlaps the promoter region of another gene. Resolution of these stalled complexes by proteasomal degradation may be necessary to allow 3′ processing factors to access the transcript or to clear the road for transcription complexes approaching the stalled complexes.

ICP27 is a multifunctional protein that interacts with a multitude of proteins, and which functions at both the transcriptional and post-transcriptional level. Its stimulation of viral transcription appears to stem from its recruitment of RNAP II to viral replication sites. Thus, ICP27 acts indirectly in transcription, that is, it recruits RNAP II. ICP27 was also found to interact with Hsc70 (Figures 2 and 3), and to be required for its redistribution to nuclear foci or VICE domains. We postulate that these foci either serve as sites where misfolded or ubiquitinated proteins are targeted and are consequently degraded, or more likely, that these foci serve as reservoirs or storage sites for the proteasomal degradation machinery, much like splicing speckles are thought to serve as storage sites for mRNA processing factors. The localization of the foci at the periphery of viral transcription-replication compartments would provide an efficient access to stalled transcription complexes, which would then be ubiquitinated and degraded. Hsc70 may even promote ubiquitination of RNAP II for degradation by the proteasome because the dominant negative mutant partly blocked this occurrence.

**Figure 11:**
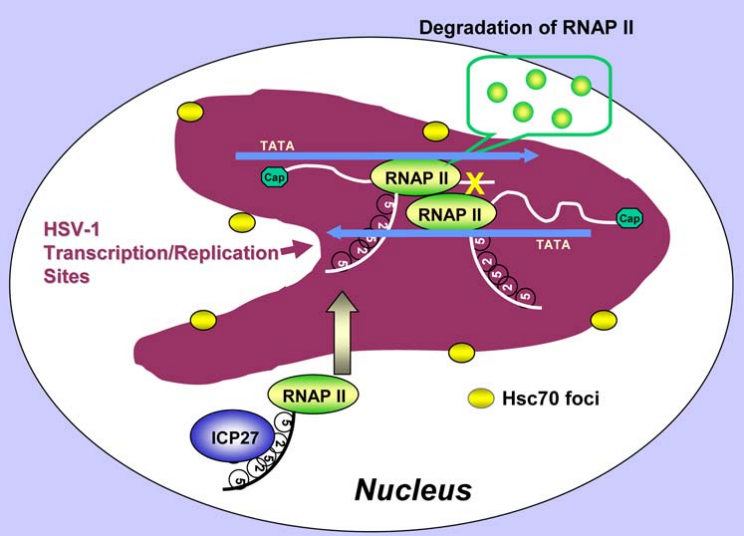
Model of RNAP II Degradation during HSV-1 Transcription. ICP27 interacts with the CTD of RNAP II [11] and recruits RNAP II to a viral replication compartment [11], [34], which is represented in magenta. Elongating RNAP II complexes on opposite DNA strands may collide causing one or both complexes to stall. At least one of the stalled complexes may then be ubiquitinated and degraded by the proteasome. The other transcription complex is now free to continue. Hsc70 foci, which form at the same time as replication compartments, and which lie at their periphery, may aid this process by providing a ready source of chaperone proteins as well as other components of the 26S proteasome [6], [7].

A model to explain the role of ICP27 in this process is shown in Figure 11. ICP27 interacts with the CTD of RNAP II early in infection and recruits RNAP II to sites of viral DNA, which develop into replication compartments [46], [47]. The HSV-1 genome encodes transcripts on both strands, as well overlapping transcripts that share 5′ or 3′ ends. Thus, during robust viral transcription, it is conceivable that elongating RNAP II complexes may collide and thus stall as depicted in Figure 11. Additionally, elongating RNAP II complexes may pile up in regions where there are several transcripts that share 3′ ends, and which may be transcribed with different kinetics. Clearance of these roadblocks is required to allow transcription to continue and to allow 3′ end formation. Components of the nuclear foci or VICE domains, including Hsc70 may then be called into action to ubiquitinate and degrade the stalled complexes. Hsc70 appears to play an important role in HSV-1 replication because when its action was blocked by the dominant negative mutant, viral yields were reduced, just as occurred with the addition of proteasome inhibitors.

These studies have uncovered yet another layer to the complexity of activities that are mediated by ICP27. In addition to its reported transcriptional and post-transcription roles, we now show that ICP27 is also involved in the resolution of stalled RNAP II complexes by proteasomal degradation through its interaction with and redistribution of Hsc70.

## Materials and Methods

### Cells, viruses and recombinant plasmids

Vero cells, HeLa R19 cells and rabbit skin fibroblasts (RSF) were grown on minimal essential medium containing 10% fetal bovine serum. Vero 2-2 cells were grown in medium containing 750 µg/ml G418 as described previously [25]. HSV-1 strain KOS and 27-LacZ were described previously [25]. ICP27 viral mutants dLeu, d1-2, d5-6, n406 and n504 were generously provided by Steve Rice, and have been described previously [18], [21], [23]. ICP27 recombinant plasmids, pΔN, pD2ΔS5, pH17, pΔC and pΔ26-100 were described [11]. Plasmids pGEX-Hsc70 (1-540), pGEX-Hsc70 (373-540), pGEX-Hsc70 (373-650) and pGEX-Hsc70 (540-650) were generously provided by Ernst Ungewickell [4]. Plasmids pcDNA3.1-Hygro(+)Myc-Hsc70 and pcDNA3.1-Hygro(+)Myc-Hsc70K71M expressing Myc-Hsc70 and Myc-Hsc70K71M were generously provided by Daniel Manor [3]. Plasmids pEGFPC3-Hsc70 and pEGFPC3-Hsc70K71M were constructed by ligating Hsc70 and Hsc70K71M cDNA in frame into pEGFC3 vector (Clonetech).

### Transfection, virus infection and immunoprecipitation procedures

Transfection of plasmid DNA was performed using Lipofectamine (Invitrogen) according to the manufacturer's protocol. Twenty-four hours after transfection, cells were infected with WT or mutant HSV-1 as indicated at a multiplicity of infection (moi) of 10. For direct infections without prior transfection, cells were infected with WT or ICP27 mutant viruses at a moi of 10. Nuclear extracts and whole cell extracts were prepared at the times indicated as described previously [26], [27], [29], [30]. Immunoprecipitations were performed as described previously [11] with anti-ICP27 monoclonal antibody H1113 (Virusys), anti-Hsc70 antibody (StressGen) and anti-ubiquitin antibody (StressGen). Gel electrophoresis and immunoblotting procedures were performed as described [11], [29], [30].

### In vitro binding assays

GST-binding assays were performed at room temperature by combining 10 µl of Glutathione-Sepharose-bound GST proteins with 10 µl of in vitro translation reaction containing ^35^S-methionine labeled WT ICP27 or mutant versions as described [27], [29]. Beads were washed four times and bound proteins were fractionated by SDS-polyacrylamide gel electrophoresis.

### Immunofluorescence microscopy

Cells were grown on coverslips and transfected and/or infected as described in the figure legends. Infections with WT or ICP27 mutants were performed at a moi of 10. At the indicated times, cells were fixed in 3.7% formaldehyde and immunofluorescent staining was performed as described previously [11], [27] with anti-ICP27 monoclonal antibodies H1119 or H1113 (Virusys), anti-ICP4 monoclonal antibody H1101 (Virusys), anti-ICP0 monoclonal antibody H1112 (Virusys), anti-ICP8 antibody H1115 (Virusys), anti-RNAP II antibodies H5 and H14 (Covance Research Products), anti-RNAP II antibody ARNA3 (Research Diagnostics), anti-Hsc70 antibody (StressGen) and anti-SC35 hybridoma supernatant [27]. Procedures for the serial treatment of cells with different monoclonal antibodies were described previously [27]. GFP fluorescence was viewed directly. Cells were viewed with a Zeiss Axiovert S100 microscope. Images were pseudocolored and merged using Adobe Photoshop.

## Supporting Information

Figure S1 Phospho-serine 5 RNAP II Colocalizes with HSV-1 Replication Compartments. Vero cells were infected with WT HSV-1, dLeu or n406 for the times indicated. Cells were stained with H14 antibody, which recognizes RNAP II phosphoserine-5 and with ICP4 to mark replication compartments (left panels). Cells were stained with H5 antibody, which recognizes the serine-2 phospho-form of RNAP II CTD, and which cross-reacts with SR protein SC35 under conditions in which serine-2 RNAP II levels are low and SR protein levels are high [11], [40]. Cells were also stained ICP4 antibody (right panels). Arrows in the left panels point to H14 (green) structures (green) that colocalize with ICP4-marked replication compartments (red) for WT infected cells at 8 h or a pre-replication site for the n406 infected cell. The arrows in the right panels show H5 staining in a speckled splicing structure (green) and ICP4 marked replication compartment (red).
(2.23 MB TIF)

Figure S2 Loss of Phospho-serine 2 Staining Does Not Occur in ICP27 Mutant d-Leu and n406 Infections. Vero cells were infected with WT HSV-1, dLeu or n406 for 8 h. Cells were stained with H5 antibody, which recognizes the serine-2 phospho-form of RNAP II CTD, and which cross-reacts with SR protein SC35 under conditions in which serine-2 RNAP II levels are low and SR protein levels are high [11], [40]. Cells were also stained with anti-SC35, and in the bottom panel, anti-Hsc70 antibody. Arrows point to an H5-SC35 splicing speckle in the top panels, and to an Hsc70 focus adjacent to an SC35 splicing speckle in the bottom merge panel.
(2.21 MB TIF)

Figure S3 Dominant Negative Mutant Hsc70K71M Does Not Interact with ICP27. A) Schematic diagram of Hsc70 showing the position of the lysine to methionine substitution in the ATPase domain of Hsc70. B) RSF cells were transfected with a GFP control plasmid or with GFP-Hsc70 or mutant GFP-Hsc70K71M. Twenty-four hours later cells were infected with WT HSV-1 for 8 h. Immunoprecipitation was performed with anti-ICP27 antibody and protein complexes were fractionated by SDS-PAGE and transferred to nitrocellulose. The Western blot was first probed with anti-ICP27 antibody (left panel). The same blot was washed and subsequently probed with anti-GFP antibody (middle panel). The arrow indicates the position of GFP-Hsc70. The blot was again washed and probed with anti-Hsc70 antibody (right panel). The arrow indicates the position of endogenous Hsc70. The bands marked with asterisks are heavy chain IgG from the immunoprecipitations.
(0.67 MB TIF)
