# Supplementary figures and images for: Hsc70 Focus Formation at the Periphery of HSV-1 Transcription Sites Requires ICP27
Source: PLoS One. 2008 Jan 30;3(1):e1491. doi: 10.1371/journal.pone.0001491 (PMC2200795; doi:10.1371/journal.pone.0001491)

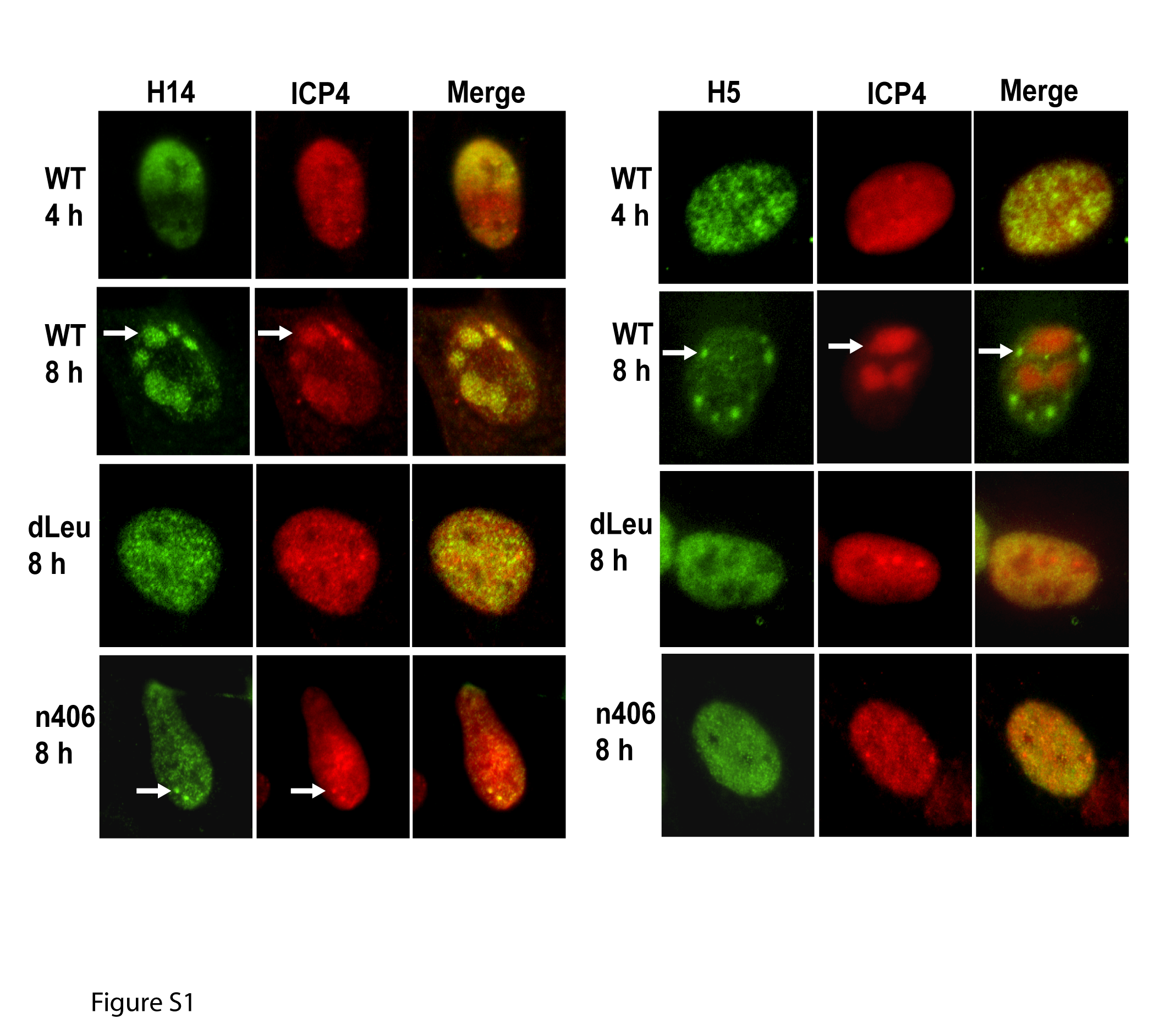

Supplement: Figure S1 — Phospho-serine 5 RNAP II Colocalizes with HSV-1 Replication Compartments. Vero cells were infected with WT HSV-1, dLeu or n406 for the times indicated. Cells were stained with H14 antibody, which recognizes RNAP II phosphoserine-5 and with ICP4 to mark replication compartments (left panels). Cells were stained with H5 antibody, which recognizes the serine-2 phospho-form of RNAP II CTD, and which cross-reacts with SR protein SC35 under conditions in which serine-2 RNAP II levels are low and SR protein levels are high [11], [40]. Cells were also stained ICP4 antibody (right panels). Arrows in the left panels point to H14 (green) structures (green) that colocalize with ICP4-marked replication compartments (red) for WT infected cells at 8 h or a pre-replication site for the n406 infected cell. The arrows in the right panels show H5 staining in a speckled splicing structure (green) and ICP4 marked replication compartment (red). (2.23 MB TIF) [file pone.0001491.s001.tif]

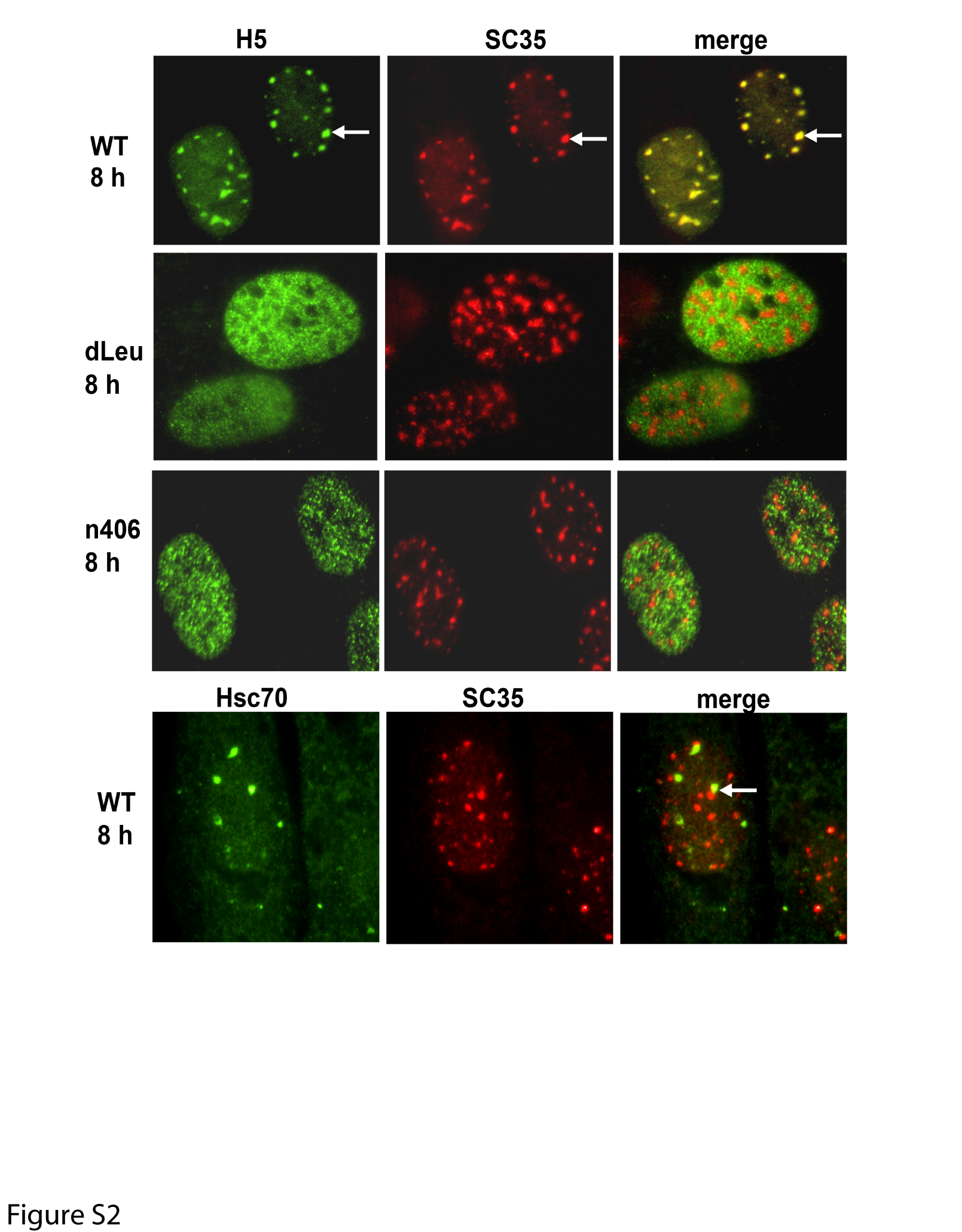

Supplement: Figure S2 — Loss of Phospho-serine 2 Staining Does Not Occur in ICP27 Mutant d-Leu and n406 Infections. Vero cells were infected with WT HSV-1, dLeu or n406 for 8 h. Cells were stained with H5 antibody, which recognizes the serine-2 phospho-form of RNAP II CTD, and which cross-reacts with SR protein SC35 under conditions in which serine-2 RNAP II levels are low and SR protein levels are high [11], [40]. Cells were also stained with anti-SC35, and in the bottom panel, anti-Hsc70 antibody. Arrows point to an H5-SC35 splicing speckle in the top panels, and to an Hsc70 focus adjacent to an SC35 splicing speckle in the bottom merge panel. (2.21 MB TIF) [file pone.0001491.s002.tif]

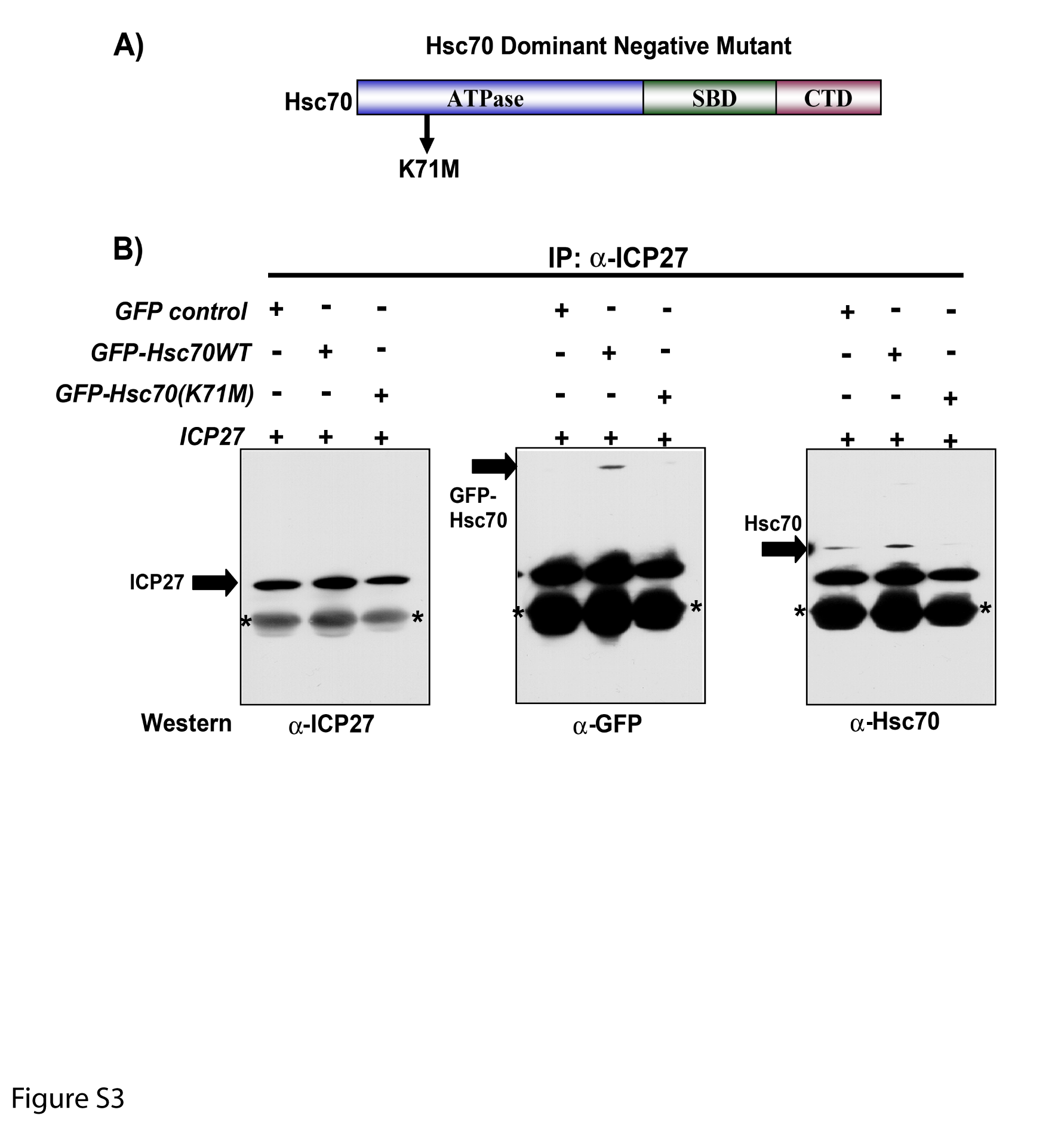

Supplement: Figure S3 — Dominant Negative Mutant Hsc70K71M Does Not Interact with ICP27. A) Schematic diagram of Hsc70 showing the position of the lysine to methionine substitution in the ATPase domain of Hsc70. B) RSF cells were transfected with a GFP control plasmid or with GFP-Hsc70 or mutant GFP-Hsc70K71M. Twenty-four hours later cells were infected with WT HSV-1 for 8 h. Immunoprecipitation was performed with anti-ICP27 antibody and protein complexes were fractionated by SDS-PAGE and transferred to nitrocellulose. The Western blot was first probed with anti-ICP27 antibody (left panel). The same blot was washed and subsequently probed with anti-GFP antibody (middle panel). The arrow indicates the position of GFP-Hsc70. The blot was again washed and probed with anti-Hsc70 antibody (right panel). The arrow indicates the position of endogenous Hsc70. The bands marked with asterisks are heavy chain IgG from the immunoprecipitations. (0.67 MB TIF) [file pone.0001491.s003.tif]
